# Modulation of NRF2/KEAP1 Signaling by Phytotherapeutics in Periodontitis

**DOI:** 10.3390/antiox13101270

**Published:** 2024-10-18

**Authors:** Giovanni Tossetta, Sonia Fantone, Lucrezia Togni, Andrea Santarelli, Fabiola Olivieri, Daniela Marzioni, Maria Rita Rippo

**Affiliations:** 1 Department of Experimental and Clinical Medicine, Università Politecnica delle Marche, 60126 Ancona, Italy; g.tossetta@univpm.it; 2 Scientific Direction, IRCCS INRCA, 60124 Ancona, Italy; s.fantone@inrca.it (S.F.); f.olivieri@univpm.it (F.O.); 3 Department of Clinical Specialistic and Dental Sciences, Università Politecnica delle Marche, 60126 Ancona, Italy; l.togni@staff.univpm.it (L.T.); andrea.santarelli@staff.univpm.it (A.S.); 4 Dentistry Clinic, National Institute of Health and Science of Aging, IRCCS INRCA, 60126 Ancona, Italy; 5 Department of Clinical and Molecular Sciences, DISCLIMO, Università Politecnica delle Marche, 60126 Ancona, Italy; m.r.rippo@univpm.it; 6 IRCCS INRCA, 60124 Ancona, Italy

**Keywords:** nuclear factor erythroid 2-related factor 2 (NRF2), oxidative stress, periodontitis, phytotherapeutics, compounds

## Abstract

Periodontitis affects up to 40% of adults over 60 years old and is a consequence of gingivitis. Periodontitis is characterized by a chronic inflammation, periodontal damage, and alveolar bone resorption. The nuclear factor erythroid 2-related factor 2 (NFE2L2 or NRF2)/Kelch-like ECH-Associated Protein 1 (KEAP1) (NRF2/KEAP1) signaling pathway plays a key role in periodontitis by modulating redox balance and inflammation of the periodontium. However, NRF2 expression is decreased in gingival tissues of patients with periodontitis while oxidative stress is significantly increased in this pathology. Oxidative stress and lipopolysaccharide (LPS) produced by gram-negative bacteria favor the production of inflammatory causing periodontal inflammation and favoring alveolar bone. In this review, we analyzed the current literature regarding the role of natural and synthetic compounds in modulating the NRF2/KEAP1 pathway in in vitro and in vivo models of periodontitis in order to evaluate new potential treatments of periodontitis that can improve the outcome of this disease.

## 1. Introduction

Periodontitis is a chronic multifactorial inflammatory disease associated with dysbiotic plaque biofilms and characterized by a progressive destruction of the tooth-supporting apparatus [1,2]. Its main clinical features include the loss of periodontal tissue, due to the clinical attachment loss and the alveolar bone loss, and the presence of periodontal pocketing and gingival bleeding [3,4]. Periodontitis represents a major public health problem that contributes to the global burden of chronic non-communicable diseases. According to the Global Burden of Disease, periodontitis was ranked as one of the most prevalent conditions of humankind between 1990 to 2010, and a recent update confirmed that its prevalence is still substantial and worrisome, with an overall prevalence equal to 61.6% [5]. Periodontitis accounts for a substantial proportion of edentulism and masticatory dysfunction, resulting in significant dental care costs and a negative impact on general health [6]. It may lead to tooth loss and instability, negatively affect chewing function and aesthetics, be a source of social inequality, and impair quality of life [6].

According to the new classification of periodontal and peri-implant diseases, stages III and IV represent the most complex cases of periodontitis, due to the presence of angular defects, furcation involvements, tooth mobility, extensive tooth loss, and loss of function. In these stages, several intrinsic or environmental risk factors adversely affect the ability of the host to respond to the bacterial infection and to contain the tissue damage. Moreover, a significantly rapid progressive damage to the attachment apparatus is appreciated. Fortunately, a relative limited proportion of the population suffers from severe periodontitis (10–12%) and, usually, only a few teeth per person are involved [7,8,9].

The main risk factors associated with the periodontitis are the smoking and the diabetes mellitus. Other modifiable and non-modifiable risk factors may contribute to the developmental of periodontal disease, such as alcohol abuse, systemic diseases (cardiovascular, immunological, metabolic, and hematological diseases, as well as malnutrition), polypharmacological therapies, stress conditions, a sedentary lifestyle, and genetic predisposition. The progression of the periodontitis seems to be dependent on an abnormal host response to sub-gingival plaque biofilm. Over the past few years, strong evidence has emerged to implicate oxidative stress or presence of specific pathogens in pathogenesis of periodontitis. Since the nuclear factor erythroid 2-related factor 2 (NFE2L2 or NRF2)/Kelch-like ECH-Associated Protein 1 (KEAP1) (NRF2/KEAP1) signaling is the main pathway involved in the regulation of cellular redox homeostasis, several studies have investigated the role of this pathway in periodontitis pathophysiology. In fact, it has been demonstrated that NRF2/KEAP1 signaling is involved in the regulation of many cell processes that are altered in periodontitis.

Dental plaque harbors several bacterial pathogens which stimulate host cells to release various pro-inflammatory cytokines, leading to the hyper production of proteolytic enzymes and O_2_ by oxidative burst [10]. *Porphyromonas gingivalis* (*P. gingivalis*), *Tannerella forsythia* (*T. forsythia*) and *Aggregatibacter actinomycetemcomitans* (*A. actinomycetemcomitans*) are the most common pathogens associated to periodontitis [11]. These periodontal pathogens are equipped with several virulence factors including fimbriae, adhesins, lipopolysaccharides, hemagglutinins, proteinases, and toxic products that favor pathogen survival and proliferation [12].

Reactive Oxygen Species (ROS) [13] play a key role in the pathophysiology of several diseases [14,15], including cancer [16,17,18,19,20], inflammatory diseases [21,22], and oral cavity diseases such as periodontitis [23,24]. The antioxidant defenses of the cell are able to mitigate the negative effects of high ROS levels, although harmful effects will take place if ROS overwhelm the antioxidant capacity of the cell [14,15,25]. Neutrophils play a key role in periodontitis since they represent the first defense line against pathogenic biofilm during periodontitis but also the major producers of ROS since they use the latter to fight the pathogenic bacteria [26]. In addition, lipopolysaccharide (LPS), produced by gram-negative bacteria during periodontitis, induces periodontal inflammation characterized by high levels of tumor necrosis factor (TNF)-α, interleukin (IL)-6, and IL-1, as well as of nuclear factor-κB ligand (RANKL) which, in turn, causes an excessive osteoclast formation and activation, leading to alveolar bone loss [27,28,29]. In fact, RANKL is produced by osteoblasts, periodontal ligament fibroblasts and inflammatory cells under pro-inflammatory stimuli and plays a key role in osteoclastogenesis [27,30]. Inflammation may also be favored by the increased blood vessels found in gingiva of patients with periodontitis [31,32,33]. However, this mechanism in periodontitis requires more evidence and it is not widely recognized.

**Figure 1:**
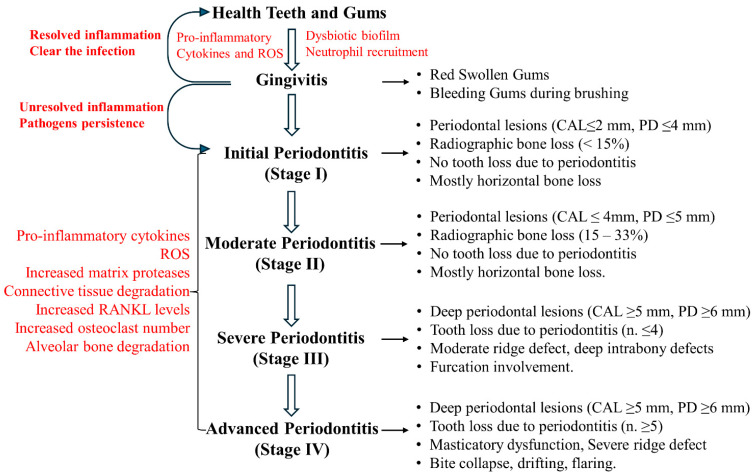
Pathogenesis of periodontitis. If gingivitis remains unresolved, there is a persistence of pathogens and inflammation that leads to different grades of periodontitis. CAL = clinical attachment loss; PD = probing depth.

A schematic representation of periodontitis pathogenesis is shown in Figure 1.

**Figure 2:**
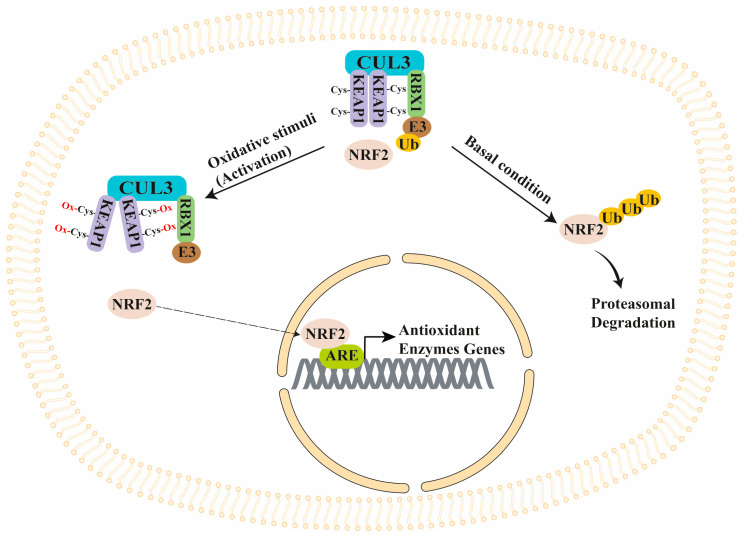
NRF2/KEAP1 signaling regulation. Under the basal condition, NRF2 is bound to the KEAP1/CUL3/RBX1 E3-Ub ligase complex that induces NRF2 proteasomal degradation. Under oxidant stimuli, ROS oxidate the cysteine residues of KEAP1, causing a conformational change that inhibits NRF2 ubiquitination/degradation. Since NRF2 avoids proteasomal degradation, it can migrate into the nucleus and bind ARE regions present in the upstream regulatory region (promoter) of several antioxidant genes, causing their transcription. ARE, antioxidant response element; Cul3, Cullin 3; E3, Ubiquitin ligase 3; KEAP1, Kelch Like ECH Associated Protein 1; NRF2, Nuclear Factor Erythroid 2-Related Factor 2; RBX1, RING box protein 1; Ub, Ubiquitin.

The nuclear factor erythroid 2-related factor 2 (NFE2L2 or NRF2)/Kelch-like ECH-Associated Protein 1 (KEAP1) (NRF2/KEAP1) signaling is a master antioxidant pathway in cells reducing ROS through the induction of genes encoding several antioxidant and phase II detoxifying enzymes [34,35]. Normally, NRF2 is present in the cytoplasm as an inactive complex and is bound to its repressor KEAP1. The latter is part of the Cullin 3 (Cul3)/RING box protein 1 (RBX1) E3-ubiquitin ligase complex that favors NRF2 ubiquitination and proteasomal degradation [35,36,37]. However, KEAP1 contains many reactive cysteine residues that act as sensors of intracellular redox conditions. In fact, modification (oxidation) of these cysteine residues (under oxidant stimuli) causes KEAP1 conformational changes that inhibit NRF2 proteasomal degradation and cause its nuclear translocation, allowing its binding to the antioxidant response elements (AREs) in the promoter of antioxidant enzyme genes, thus inducing their transcription [35,37,38] (Figure 2).

The multifaceted role of NRF2/KEAP1 signaling has been widely demonstrated in several cancerous [20,35,37] and non-cancerous [34,36,39,40,41] diseases, including periodontitis [42,43]. It is known that NRF2 expression is decreased in gingival tissues of patients with severe periodontitis [44] and, accordingly, that increased ROS levels during periodontitis worsens periodontal inflammation [45].

The effects of the NRF2/KEAP1 pathway have also been proven in NRF2 knockdown mice model of periodontitis where NRF2 absence caused a more severe alveolar bone loss as well as an increased oxidative stress in periodontal tissue [46]. The important role of this signaling has also been demonstrated in human periodontal ligament stem cells (hPDLSCs) where NRF2 overexpression attenuated apoptosis in oxidative stress conditions by activating the expression of antioxidant enzymes [47].

The NRF2/KEAP1 signaling pathway plays a key role also in osteoclastogenesis since mouse macrophages exposed to RANKL showed a decreased expression of NRF2 and NRF2-dependent antioxidant enzymes such as Heme-oxygenase 1 (HO-1) and NAD(P)H:quinone oxidoreductase (NQO1) [48]. These data demonstrated that NRF2 is an important inhibitor of osteoclastogenesis. In fact, it has been found that NRF2 overexpression induced the expression of the antioxidant enzymes HO-1, γ-glutamylcysteine synthetase (GCS) and NQO1 reducing osteoclast differentiation and then bone destruction [48].

Looking at the beneficial effects of NRF2/KEAP1 signaling activation reported in several studies, it is reasonable to think that increasing NRF2 expression in oral cavity could be an efficient therapeutic strategy for the prevention or treatment of patients with periodontitis.

The purpose of this review is to explore the potential role of natural compounds to promote NRF2/KEAP1 signaling activation by in vitro and in vivo models of periodontitis in order to suggest the use of these compounds to prevent or treat periodontitis, thus preventing or improving the outcome of this disease.

## 2. NRF2/KEAP1 Signaling Activation by Phytotherapeutics in Periodontitis Models

Natural compounds are biological substances used by plants (e.g., polyphenols, carotenoids, flavonoids, and anthocyanins) to protect themself from predators or external influences. Natural compounds can also be isolated from bacteria, fungi, and marine organisms [35,38,49,50,51]. Several natural compounds have shown important beneficial effects in many diseases, often for their antioxidant and anti-inflammatory effects, and are therefore used as supplements in natural medicine [35,38,49,50]. For these reasons, these compounds could also be used as supplement to protect oral tissues from oxidative stress and inflammation during periodontitis. Moreover, the presence of these compounds in chewing sticks, used as tools for oral hygiene, may show significant beneficial effects in preventing/treat periodontitis.

The studies discussed in this section are summarized in Table 1.

*Quercetin* is a natural compound with important antioxidant effects that can be found in many fruits and vegetables [52]. It has been reported that quercetin treatment of H_2_O_2_-exposed human periodontal ligament cells (hPDLCs) increased NRF2, NQO1, catalase (CAT), and HO-1 expression, reducing ROS, DNA damage, and cellular senescence. Quercetin also favored osteogenesis in H_2_O_2_-exposed hPDLCs. Moreover, quercetin treatment of periodontitis in mice increased NRF2 and SOD expression while reduced alveolar bone loss. Thus, quercetin can significantly improve antioxidant status and alveolar bone loss in periodontitis [53].

*Biochanin A* (BA) is an isoflavone present in several herbal products and has important anti-inflammatory and antioxidant effects [54]. Zhang et al. evaluated the effects of BA in rats with experimental periodontitis and found that BA treatment alleviated alveolar bone resorption and reduced interleukin (IL)-1β, Tumor Necrosis Factor (TNF)-α and ROS levels, as well as increased NRF2 protein expression demonstrating that BA can inhibit inflammation and bone loss in periodontitis [55].

*Curcumin* is a natural polyphenolic phytochemical widely used for its antioxidant, anticancer, and anti-inflammatory effects [49,56,57,58]. It can also reduce bone loss, inhibiting the proliferation and differentiation of osteoclasts and promoting their apoptosis [59,60]. A key role in osteogenic differentiation is played by the PI3K/AKT signaling pathway since it can favor this process [61,62]. An interesting study found that curcumin promoted osteogenic differentiation of human periodontal ligament stem cells (hPDLSCs) and activated the PI3K/AKT/NRF2 signaling pathway, favoring AKT phosphorylation and inducing NRF2 expression and its nuclear translocation. Interestingly, the inhibition of the PI3K/AKT signaling with the inhibitor LY294002 significantly decreased NRF2 expression. Moreover, the silencing of NRF2 (by siRNA) significantly reversed curcumin-induced osteogenic differentiation of hPDLSCs [63]. Thus, curcumin can induce the osteogenesis modulating PI3K/AKT/NRF2 signaling pathway. Additionally, curcumin pretreatment of an H400 oral epithelial cell line exposed to *Fusobacterium nucleatum* reduced IL-1β, TNF-α, and IL-8 expression while increasing NRF2 and HO-1 expression [64].

*10-oxo-trans-11-octadecenoic acid* (KetoC) is a bioactive metabolite generated from linoleic acid (abundant in sunflower seeds, walnuts, soybeans, corn, olives, and their oils) by intestinal microorganisms with important antioxidant and anti-inflammatory effects [65,66]. It has been found that KetoC treatment of gingival epithelial cells (GECs) increased the expression of NRF2, HO-1, and NQO1, decreasing ROS levels and demonstrating a protective function against the oxidative stress [67].

*Caffeic acid phenethyl ester* (CAPE) is a natural compound found in several plants that has important anti-inflammatory effects on periodontal inflammation [68,69]. It has been reported that primary murine macrophages, RAW 264.7 cells and primary human gingival fibroblasts exposed to CAPE showed an increased HO-1 expression. Moreover, NRF2 silencing attenuated CAPE-induced HO1 expression in macrophages and CAPE reduced IL-1α and IL-1β levels in primary murine macrophages and RAW 264.7 cells exposed to periodontal pathogens. Blocking HO-1 by its specific inhibitor (SnPP) decreased the antioxidative activity and attenuated the anti-inflammatory activity of CAPE. Thus, CAPE exerted its antioxidant and anti-inflammatory effects through the modulation of NRF2/HO-1 pathway [70].

*Paeonol* is a natural phenolic compound isolated from the root bark of *Paeonia suffruticosa Andrews*, a shrub widely used in traditional Chinese herbal medicine for its important anti-inflammatory and antioxidant activity [71,72]. Li et al. showed that paeonol treatment of periodontitis-induced rats decreased RANKL expression inhibiting osteoclasts formation. Moreover, paeonol reduced ROS, pro-inflammatory cytokines levels (IL-1β, IL-6 and TNF-α), and NF-κB activation, and alleviated oxidative stress, increasing HO-1 expression and glutathione (GSH) levels in gingival tissues. Importantly, paeonol increased NRF2 expression while NRF2 silencing decreased the inhibitory effect of paeonol on NF-κB activation, suggesting that the protective effect of paeonol against periodontitis-induced osteoclastogenesis and alveolar bone loss is mediated by the regulating of the NRF2/NF-κB signaling pathway [73].

*Euphorbia factor L1* (EFL1) is a diterpenoid isolated from *Euphorbia lathyris* with several beneficial effects [74]. It has been reported that EFL1 treatment of mouse bone marrow-derived macrophages (used as osteoclast precursor) suppressed osteoclast formation and bone resorption inhibiting RANKL-induced c-Fos expression. Moreover, EFL1 decreased ROS levels, activating NRF2 signaling and increasing the expression of sulfiredoxin (SRX), peroxiredoxins (PRXs), and thioredoxins (TRXs). EFL1 also induced apoptosis in differentiated osteoclasts and inhibited inflammation-induced bone erosion in mice, suggesting that EFL1 regulates osteoclast differentiation by modulating redox status and inducing apoptosis in osteoclasts [75].

*Resveratrol *is a stilbenoid with important antioxidant and anti-inflammatory properties that can be found in grape, blueberries, raspberries, and mulberries [76]. Tamaki et al. showed that resveratrol could prevent the progression of periodontitis and reduce systemic oxidative stress. In fact, resveratrol administration to rats with periodontitis relieved alveolar bone resorption and activated the sirtuin 1 (SIRT1)/AMP-activated protein kinase (AMPK) and the NRF2 pathways in inflamed gingival tissues. Furthermore, resveratrol decreased TNF-α, IL-1, and IL-6 levels in rats with periodontitis [77]. In a mouse model of periodontitis, resveratrol reduced alveolar bone loss and oxidative stress in the periodontium. Interesting, NRF2 knockout reversed these results, demonstrating the involvement of NRF2 in periodontal bone healing in periodontitis [78].

*Sulforaphane* (SFN), a natural product found in cruciferous vegetables, increased intracellular reduced the glutathione (GSH)/oxidized glutathione (GSSG) ratio and the neutrophil respiratory burst in primary neutrophils from patients with periodontitis and controls. Interestingly, the chronic inflammation found in periodontitis is mainly due to the production of high levels of reactive oxygen species by neutrophils [79]. Moreover, SFN increased the expression of NRF2, NQO1, glutamate cysteine ligase catalytic (GCLC), and modifier (GCLM) subunits while reduced extracellular O_2_ (. -) production [80]. An important antioxidant effect of SFN has also been reported in gingival epithelial cells (GECs). In fact, SFN treatment of GECs significantly increased NRF2 and HO-1 expression [81].

**Table 1:** NRF2 modulators in periodontitis models.

Modulator	Structure	Model Used	Results	Ref.
Quercetin	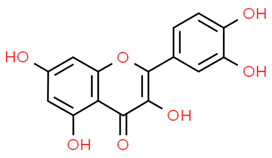	H_2_O_2_-exposed hPDLCs Periodontitis mice model	Quercetin treatment increased NRF2, NQO1, CAT, and HO-1 expression, reducing ROS, DNA damage, and cellular senescence. Quercetin increased NRF2 and SOD expression, favored osteogenesis, and reduced alveolar bone loss.	[53]
Biochanin A (BA)	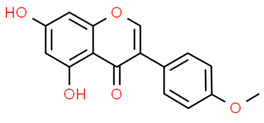	Periodontitis rat model	BA alleviated alveolar bone resorption and reduced IL-1β, TNF-α, and ROS levels, as well as increased NRF2 protein expression	[55]
Curcumin	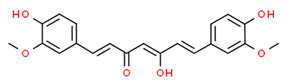	hPDLSCs	Curcumin induced AKT phosphorylation, NRF2 expression, and nuclear translocation. Inhibition of PI3K/AKT signaling decreased NRF2 expression while NRF2 silencing reversed curcumin-induced osteogenic differentiation.	[63]
		*F. nucleatum*-exposed H400 cell line	Curcumin reduced IL-1β, TNF-α, and IL-8 expression while increasing NRF2 and HO-1 expression.	[64]
10-oxo-trans-11-octadecenoic acid (KetoC)	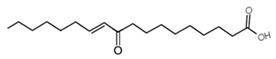	GECs	KetoC increased the expression of NRF2, HO-1, and NQO1, thus decreasing ROS levels.	[67]
Caffeic acid phenethyl ester (CAPE)	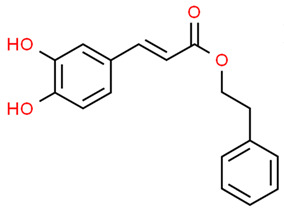	Primary murine macrophages RAW 264.7 cells Primary human gingival fibroblasts	CAPE increased HO-1 expression and reduced IL-1α and IL-1β levels. NRF2 silencing attenuated CAPE-induced HO-1 expression in macrophages. Inhibition of HO-1 by SnPP decreased the antioxidative activity and attenuated the anti-inflammatory activity of CAPE.	[70]
Paeonol	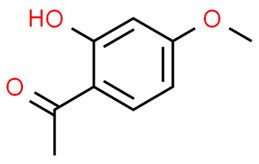	Periodontitis rat model	Paeonol decreased RANKL and inhibited osteoclasts formation. Paeonol reduced IL-1β, IL-6, and TNF-α, increased HO-1 expression and GSH levels, and reduced ROS levels in gingival tissues. Paeonol increased NRF2 expression while NRF2 silencing favored NF-κB activation by increasing pp65 subunit phosphorylation, thus abrogating the anti-inflammatory effect of paeonol.	[73]
Euphorbia factor L1 (EFL1)	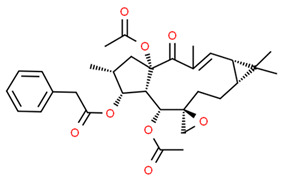	Mice bone marrow-derived macrophages (used as osteoclast precursor).	EFL1 treatment suppressed osteoclast formation and bone resorption, inhibiting RANKL-induced c-Fos expression. EFL1 decreased ROS levels activating NRF2 signaling and increasing SRX, PRXs, and TRXs expression. EFL1 induced apoptosis in differentiated osteoclasts and inhibited inflammation-induced bone erosion in mice.	[75]
Resveratrol	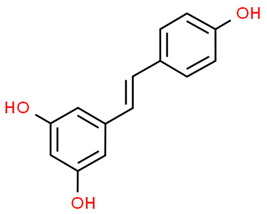	Periodontitis rat model	Resveratrol administration relieved alveolar bone resorption and activated the Sirt1/AMP-activated protein kinase (AMPK) and the NRF2 pathways in inflamed gingival tissues. Resveratrol decreased TNF-α, IL-1, and IL-6 levels.	[77]
		Periodontitis mouse model.	Resveratrol reduced alveolar bone loss and oxidative stress in the periodontium. NRF2 knockout reversed resveratrol effects.	[78]
Sulforaphane (SFN)	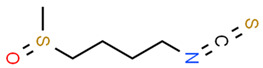	Differentiated HL60 cells (as a neutrophil model) Primary neutrophils from patients.	SFN increased the intracellular GSH/GSSG ratio and reduced the neutrophil respiratory burst. SFN increased the expression of NRF2, NQO1, GCLC, and GCLM.	[80]
		GECs	SFN increased NRF2 and HO-1 expression	[81]

hPDLCs (human periodontal ligament cells); PDLSCs (human periodontal ligament stem cells); GECs (gingival epithelial cells). The chemical structures of the compounds illustrated in this table have been taken from ChemSpider free database (https://www.chemspider.com (accessed on 30 September 2024)).

### 2.1. Effects of NRF2 Activation on RANKL-Induced Osteoclastogenesis in Periodontitis

The presence of LPS during periodontitis induces the production of pro-osteoclastogenetic cytokines such as TNF-α, IL-1, IL-6 by osteoblasts and periodontal ligament fibroblasts, T cells, and B cells, leading to the production RANKL, which has a pivotal function in osteoclast differentiation [27].

*Dehydrocostus lactone* (DL) is a natural sesquiterpene lactone derived from medicinal plants such as *Inulahelenium L.* and *Saussurea lappa* with antioxidant and anti-inflammatory properties [82,83,84,85]. Lee et al. found that RANKL-stimulated RAW 264.7 cells treated with DL attenuated NR-κB activation while increased NRF2, NQO1, sulfiredoxin (SRX), and peroxiredoxin-1 (PRX1) expression reducing ROS levels. Interestingly, NRF2 silencing promoted osteoclast differentiation, suggesting that DL attenuates osteoclast differentiation modulating NRF2 and NF-κB signaling pathways [86].

*Hesperetin* is a flavanone glycoside with anti-inflammatory and antioxidant activity that can be found in citrus fruits, grapefruits, and lemons [87]. It has been reported that hesperetin could be a potential therapeutic compound for periodontitis since it suppressed RANKL-induced osteoclastogenesis, osteoclastic bone resorption, and the activation of NF-κB and MAPK signaling in RAW 264.7 cells. Moreover, hesperetin increased NRF2, HO-1, and NQO1 expression scavenging ROS [88].

Thus, the activation of NRF2/KEAP1 signaling can significantly inhibit RANKL-induced osteoclast formation reducing inflammation and alveolar bone resorption.

**Figure 3:**
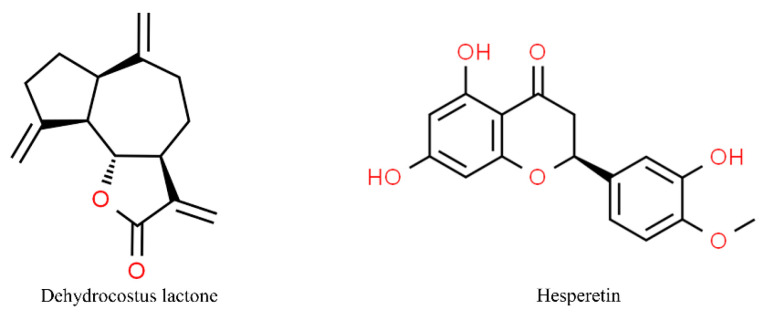
Molecular structures of dehydrocostus lactone and hesperetin. The chemical structures of the compounds illustrated in this table have been taken from ChemSpider free database (https://www.chemspider.com (accessed on 30 September 2024)).

The molecular structures of dehydrocostus lactone and hesperetin are shown in Figure 3.

### 2.2. Role of NRF2 Activation in LPS-Exposed Animal and Cell Models of Periodontitis

LPS released from gram-negative bacteria such as *P. gingivalis* stimulates ROS production from periodontal tissue, favoring the production of inflammatory cytokines such as TNF-α, IL-1, and IL-6 by inflammatory cells and causing periodontal inflammation and alveolar bone loss due to an excessive osteoclast formation and activation, since these cytokines also exert pro-osteoclastogenetic effects [27,89,90,91].

In this section, we discuss the effects of natural compounds on NRF2 activation in LPS-exposed animal and cell models of periodontitis.

The studies discussed in this section are summarized in Table 2.

*Notopterol* is a type of furanocoumarin isolated from *Notopterygium incisum* with important anti-inflammatory activities [92]. This compound may exert important function in periodontitis since notopterol treatment of LPS-stimulated human gingival fibroblasts (HGFs) significantly decreased IL-1β, IL-32, and IL-8 levels by inhibiting the activation of the NF-κB signaling pathway (inhibiting p65 subunit phosphorylation), a known pro-inflammatory signaling pathway [93]. Moreover, notopterol increased AKT and PI3K phosphorylation, as well as NRF2 expression. Notopterol also increased the expression of antioxidant enzymes such as HO-1, NQO1, CAT, and glutathione reductase (GSR), thus decreasing ROS levels. These effects were attenuated by the AKT inhibitor MK-2206.

Thus, notopterol relieved periodontal inflammation by inhibiting the activation the NF-κB and exerted antioxidant capacity activating the NRF2 and PI3K/AKT signaling pathways [94]. It is interesting to note that, according to other studies [95], the increased NRF2 expression was mediated by PI3K/AKT signaling since these effects were inhibited by MK-2206.

*Isorhamnetin* is a flavonoid isolated from *Hippophae fhamnoides* L. fruit with known anti-inflammatory effects. It has been reported that isorhamnetin treatment attenuated LPS-induced release of nitric oxide (NO), prostaglandin E2 (PGE2), IL-6, and IL-8 in human gingival fibroblasts (HGFs). Moreover, isorhamnetin inhibited LPS-induced activation of NF-κB (inhibiting p65 subunit phosphorylation), while increasing the expression of NRF2 and its downstream target HO-1. Importantly, NRF2 silencing, using siRNA, reversed the anti-inflammatory effects of isorhamnetin, suggesting that isorhamnetin inhibited LPS-induced inflammation in HGFs through the activation of the NRF2 signaling pathway [96]. This mechanism of action may be explained by the fact that silencing NRF2 decreased HO-1 expression, which inhibits NF-κB activation (a key player in inflammatory response), therefore favoring inflammation. This inhibitory effect of HO-1 on NF-κB activation may be due to the products derived from HO-1 activity such as CO, as reported in literature [97].

*Magnolol *is the main bioactive compound of *Magnolia officinalis,* a Chinese medicinal herb, and has important antioxidant and anti-inflammatory effects [98,99]. It has been found that magnolol treatment of RAW 264.7 macrophages exposed to LPS of *P. gingivalis* significantly reduced LPS-induced inflammation (reduced TNF-α, IL-1β levels), NF-κB activation, and increased NRF2 and HO-1 expression. Interestingly, NRF2/HO-1 activation by magnolol was decreased by blocking p38 MAPK activity with the specific inhibitor SB203580. This effect can be explained by the fact that MAPKs (p38, ERK, and JUK) can stimulate HO-1 expression by phosphorylating NRF2, thus favoring NRF2 nuclear translocation [100]. Inhibiting HO-1 activity by the SnPP inhibitor reversed the anti-inflammatory effects of magnolol proving that magnolol inhibits *P. gingivalis* LPS-induced inflammation in macrophages activating the NRF2/HO-1 axis, suggesting a possible use of magnolol in treatment of periodontitis [101].

*Resveratrol*, whose beneficial effects have been already discussed in the paragraph 2, also showed important effects against LPS exposure. In fact, it has been demonstrated that resveratrol treatment of LPS-stimulated human gingival fibroblasts (hGFs) reduced the expression of cyclooxygenase-2 (COX-2), matrix metalloproteinase (MMP)-2, MMP-9, and Toll-like receptor-4 (TLR4). Moreover, resveratrol reduced the activation of the MAPK signaling pathway while activating the NRF2/HO-1 axis, reducing ROS levels. Finally, resveratrol protected a periodontitis rat model against alveolar bone loss, inhibiting inflammation and osteoclast formation and increasing NRF2 and HO-1 expression in the gingiva of rat periodontitis [102]. Ma et al. confirmed these results, demonstrating that resveratrol treatment of LPS-stimulated hPDLSCs reduced IL-1β and IL-6 levels by reducing NF-κB activation (by decreasing p65/p50 subunits nuclear translocation and reducing p50 subunit expression) and increasing NRF2 and HO-1 expression. Moreover, resveratrol favored osteogenic differentiation of LPS-stimulated hPDLSCs [103]. Thus, oral administration of resveratrol may prevent the progression of periodontitis.

*Lindenenyl acetate* (LA) is one of the major constituents of *Lindera strychnifolia* Vill. (Lauraceae), a shrub native of Southeast Asia, used for its antioxidant and anti-diabetic properties [104,105]. Jeong et al. found that LA treatment of LPS-stimulated hPDLCs inhibited LPS-induced inducible nitric oxide synthase (iNOS), nitric oxide (NO), cyclooxygenase-2 (COX-2), and prostaglandin E2 (PGE2) production. LA also attenuated the production of LPS-induced TNF-α, IL-1β, IL-6, and IL-12. Moreover, LA increased HO-1 expression and enzyme activity. Pretreatment with the HO-1 inhibitor (SnPP) decreased the inhibitory activities of LA on LPS-induced PGE2, IL-1β, TNF-α, IL-6, and IL-12 production. Interestingly, the authors found that the increased expression of HO-1 was due to the activation (nuclear translocation) of NRF2. Notably, the same authors found that LA up-regulated the levels of phosphorylated c-Jun N-terminal Kinase (JNK) while JNK pathway inhibition abolished LA-induced HO-1 expression by the synthetic inhibitor SP600125. Thus, the anti-inflammatory activity of LA in HPDL cells were mediated by the HO-1, JNK, and NRF2 pathways, suggesting LA as a potential therapeutic agent in periodontal disease [106].

Macrophages play a key role in periodontal lesions, regulating the production of pro-inflammatory cytokines involved in tissue and bone destruction during periodontitis [107]. *Schisandrin* is a natural compound isolated from the dried fruits of *Schisandra chinensis* [108,109] that has shown important anti-inflammatory effects on RAW 264.7 macrophages stimulated with LPS from *P. gingivalis*. In fact, schisandrin significantly inhibited the secretion of LPS-induced proinflammatory cytokines such as TNF-α, IL-1β, and IL-6, suppressing the activation of NF-kB (by inhibiting p65 subunit expression). HO-1 inhibition (by SnPP or siRNA) inhibited the anti-inflammatory activity of schisandrin. Furthermore, schisandrin induced HO-1 expression by increasing the expression of NRF2, PI3K/Akt, and ERK activation [110]. Thus, in addition to its antioxidant effects, HO-1 can be considered also a potent anti-inflammatory molecule able to regulate pro-inflammatory mediators. The anti-inflammatory effects of schisandrin may therefore be helpful for preventing/treating periodontitis.

*Sappanchalcone* is a natural flavonoid isolated from *Caesalpinia sappan* L., a plant used in traditional Chinese medicine. It has been demonstrated that sappanchalcone exerts important anti-inflammatory and neuroprotective effects [111,112]. An interesting study reported that sappanchalcone treatment of human dental pulp cells (HDPCs) and human periodontal ligament cells (hPDLCs) increased HO-1 expression and enzyme activity in both HDPCs and hPDLCs. Moreover, sappanchalcone protected HDPCs from H_2_O_2_-induced cytotoxicity and ROS production. In addition, these authors demonstrated that the cytoprotective effect of sappanchalcone was due to the inhibition of LPS-stimulated NO, PGE2, IL-1β, TNF-α, IL-6, and IL-12 release. These anti-inflammatory effects were partly inhibited by SnPP, a specific inhibitor of HO-1. Interestingly, the authors proved that the increased HO-1 expression was due to the activation of NRF2 and c-Jun NH2-terminal kinase (JNK). Thus, sappanchalcone protected HDPCs and hPDLCs from oxidative stress and inflammation activating NRF2 pathway and JNK suggesting a potential use as therapeutic compound for periodontal, pulpal, and periapical inflammatory lesion [113].

**Table 2:** NRF2 modulators in LPS-exposed models of periodontitis.

Modulator	Structure	Model Used	Results	Ref.
Notopterol	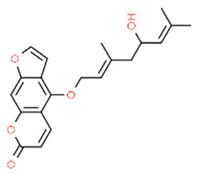	LPS-stimulated HGFs	Notopterol decreased IL-1β, IL-32, and IL-8 levels by inhibiting the activation of the NF-κB signaling pathway inhibiting the phosphorylation of p65 subunit. Notopterol increased AKT and PI3K phosphorylation and NRF2 expression. Notopterol increased HO-1, NQO1, CAT, and GSR expression and decreased ROS levels. These effects were attenuated by the AKT inhibitor MK-2206.	[94]
Isorhamnetin	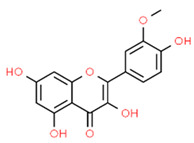	LPS-stimulated HGFs	Isorhamnetin attenuated LPS-induced release of PGE2, NO, IL-6, and IL-8, and inhibited NF-κB activation by inhibiting the phosphorylation of p65 subunit. Isorhamnetin increased the expression of NRF2 and HO-1. Silencing of NRF2 reversed the anti-inflammatory effects of isorhamnetin.	[96]
Magnolol	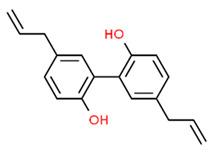	LPS-stimulated RAW 264.7	Magnolol reduced TNF-α and IL-1β levels and NF-κB activation (by inhibiting the phosphorylation of p65 subunit) while increasing NRF2 and HO-1 expression. NRF2/HO-1 activation by magnolol was diminished by blocking p38 MAPK activity. Inhibiting HO-1 activity by SnPP reversed the anti-inflammatory effects of magnolol.	[101]
Resveratrol	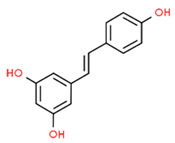	LPS-stimulated HGFs and Rats with periodontitis	Resveratrol reduced COX2, MMP-2, MMP-9, TLR4 expression and the activation of MAPK signaling pathway while activating the NRF2/HO-1 axis, reducing ROS levels. Resveratrol protected a periodontitis rat model against alveolar bone loss, inhibiting inflammation and osteoclast formation and increasing NRF2 and HO-1 expression in the gingiva of rats.	[102]
		LPS-stimulated hPDLSCs	Resveratrol reduced IL-1β and IL-6 levels and NF-κB activation by decreasing p65/p50 subunits nuclear translocation and p50 subunit expression. Resveratrol increased NRF2 and HO-1 expression and favored osteogenic differentiation.	[103]
Lindenenyl acetate (LA)	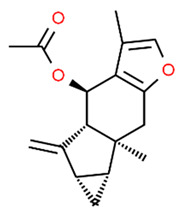	LPS-stimulated hPDLCs	LA inhibited LPS-induced iNOS, NO, COX-2, and PGE2 production, and attenuated TNF-α, IL-1β, IL-6, and IL-12 secretion. LA increased NRF2 nuclear translocation and HO-1 expression and activity. HO-1 inhibition by SnPP decreased the inhibitory activities of LA on LPS-induced inflammatory cytokines production.	[106]
Schisandrin	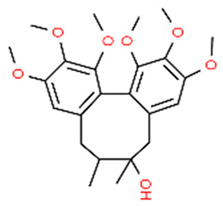	LPS-stimulated RAW 264.7	Schisandrin blocked the production of LPS-induced TNF-α, IL-1β, and IL-6, suppressing the activation of NF-kB signaling by inhibiting p65 subunit expression. Moreover, Schisandrin increased the expression of HO-1 and NRF2 and activated PI3K/Akt and ERK. Inhibiting HO-1 activity by SnPP reversed the surfactin-mediated inhibition of pro-inflammatory cytokines.	[110]
Sappanchalcone	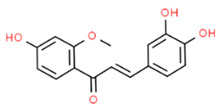	LPS-stimulated HDPCs and hPDLCs	Increased HO-1 expression and enzyme activity in both HDPCs and hPDLCs protected them from H2O2-induced ROS production. Sappanchalcone inhibited LPS-stimulated NO, PGE2, IL-1β, TNF-α, IL-6, and IL-12 release. The increased HO-1 expression was due to the activation of NRF2 and JNK.	[113]

hPDLCs (human periodontal ligament cells); HGFs (human gingival fibroblasts); human dental pulp (HDPCs); human periodontal ligament stem cells (hPDLSCs). The chemical structures of the compounds illustrated in this table have been taken from ChemSpider free database (https://www.chemspider.com (accessed on 30 September 2024)).

## 3. Role of NRF2 as Anti-Pyroptotic Target in Periodontitis

Pyroptosis is a physiological pro-inflammatory programmed death and the NOD-like receptor protein 3 (NLRP3) inflammasome pathway plays a key role in this process. NLRP3 is activated by external pathogens and danger signals, recruiting both apoptosis-associated speck-like protein containing a caspase-recruitment domain (ASC) and caspase-1 to form the NLRP3 inflammasome. Once activated, the NLRP3 inflammasome induces caspase-1 activation and the secretion of pro-inflammatory cytokines such as IL-1β and IL-18, leading to cell death [114]. The NLRP3 inflammasome can also be activated by ROS and plays a key role in inflammatory diseases including periodontitis [114]. In fact, it has been demonstrated that NLRP3-mediated pyroptosis can induce inflammation, osteoclastogenesis, and alveolar bone loss [115].

The studies discussed in this section are summarized in Table 3.

*Kynurenic acid* (KA) is a metabolite of tryptophan, an amino acid present in many protein-based foods such as almonds [116,117], with important anti-inflammatory potential. It has been reported that KA treatment significantly improved the LPS-induced THP-1 macrophage viability, preventing pyroptosis through the reduction of NLRP3 and Caspase-1 expression, as well as IL-1β, IL-18, and TNF-α levels. Other authors have demonstrated that KA suppressed the NLRP3 inflammasome activation through the activation of NRF2/HO-1 axis, which led to ROS inhibition. These anti-pyroptotic and antioxidant effects of KA could be reversed by the inhibition of NRF2 (by using the synthetic inhibitor ML385). These data demonstrate that KA exerts its anti-pyroptotic effects through the activation of the NRF2 pathway [118].

In addition to KA, four other compounds (epigallocatechin-3-gallate, silibinin, chlorogenic acid, and eldecalcitol) have been found to be involved in the modulation of the NRF2/HO-1/NLRP3 axis. 

*Epigallocatechin-3-gallate* (EGCG) is a polyphenol contained in green tea, with important anti-bacterial, anti-inflammatory, and antioxidant capacity [119]. It has been reported that EGCG significantly reduced alveolar bone loss in a periodontitis rat model. Moreover, EGCG decreased IL-1β, IL-18, TNF-α levels, decreasing NLRP3 expression and NF-κB activation. Additionally, the authors showed that EGCG increased NRF2, HO-1 and SOD expression decreasing oxidative stress [120].

*Silibinin* (SB) is a natural compound that can be found in silymarin, also called milk thistle or *Silybum marianum,* with important antioxidant and anti-inflammatory effects [121]. Li et al. found that SB reduced alveolar bone loss, oxidative stress, NF-κB and NLRP3 expression, and TNF-α, IL-1β, and IL-6, while increasing NRF2 expression in the periodontitis rat model. Thus, SB exhibited important anti-inflammatory and antioxidative properties by regulating both NRF2 and NF-κB signaling, suggesting a promising potential clinical application of this compound in treatment of periodontitis [122].

*Chlorogenic acid* (CA) is a natural compound isolated from *Coffea canephora*, *Coffea arabica* L. and *Lonicerae japonicae* with anticancer, antioxidant and anti-inflammatory effects [123,124,125]. It has been found that CA treatment of LPS-induced Human gingival fibroblasts (HGFs) inhibited NLRP3 expression and reduced IL-1β and IL-18 levels while increasing NRF2 and HO-1 expression and reducing oxidative stress, suggesting that CA could attenuate inflammation in HGFs during periodontitis [126]. These results are in agreement with another study that evaluated the effects of CA in LPS-induced immortalized human oral keratinocytes (IHOKs). In this study, the authors found that CA treatment attenuated LPS-induced inflammatory mediators release (e.g., PGE2), ROS production, and NF-κB activation, reducing inflammation. Moreover, CA promoted NRF2 translocation and HO-1 expression. Thus, coffee consumption may be beneficial for alleviating periodontitis [127].

**Table 3:** Modulators targeting NRF2 to inhibit pyroptosis in periodontitis.

Modulator	Structure	Model Used	Results	Ref.
Kynurenic acid (KA)	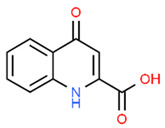	LPS-induced THP-1 macrophage	KA improved cell viability preventing pyroptosis through the reduction of NLRP3 and Caspase-1 expression, as well as IL-1β, IL-18, and TNF-α levels. KA suppressed NLRP3 inflammasome activation through the activation of the NRF2/HO-1 axis, which led to ROS inhibition. These effects were reversed by the inhibition of NRF2.	[118]
Epigallocatechin-3-gallate (EGCG)	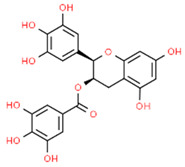	Periodontitis rat model	EGCG reduced alveolar bone loss, lowered IL-1β, IL-18, TNF-α levels, and decreased NLRP3 expression and NF-κB activation (inhibiting p65 subunit expression). EGCG increased NRF2 and HO-1 expression, decreasing oxidative stress.	[120]
Silibinin (SB)	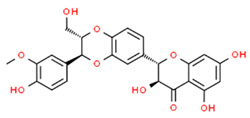	Periodontitis rat model	SB reduced alveolar bone loss, oxidative stress, NF-κB (inhibiting p65 subunit expression), NLRP3 expression, and TNF-α, IL-1β, and IL-6 levels while increasing NRF2 expression in the periodontium.	[122]
Chlorogenic acid (CA)	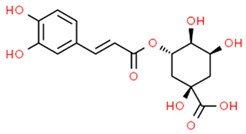	LPS-induced HGFs	CA treatment inhibited the contents of IL-1β and IL-18 while increasing NRF2 and HO-1 expression, reducing oxidative stress.	[126]
		LPS-induced IHOKs	CA treatment attenuated LPS-induced inflammatory mediators release (PGE2), ROS production, and NF-κB activation (by inhibiting p65 subunit phosphorylation), reducing inflammation. Moreover, CA promoted NRF2 translocation and HO-1 expression.	[127]

HGFs (human gingival fibroblasts); IHOKs (immortalized human oral keratinocytes). The chemical structures of the compounds illustrated in this table have been taken from ChemSpider free database (https://www.chemspider.com (accessed on 30 September 2024)).

## 4. Role of NRF2 in Periodontitis Complicated by Diabetes

Diabetes mellitus (DM) is a complex metabolic disorder characterized by an impaired glucose tolerance and hyperglycemia as a result of insulin deficiency or resistance [128]. Chronic hyperglycemia can result in a damage of several organs and tissues, including the retina, kidneys, heart, and blood vessels. Moreover, DM has been associated with an increased risk of developing cardiovascular disease (CVD) [129,130].

DM is a well-recognized risk factor of periodontitis. It is formally included in the grading classification system of periodontitis because the prevalence and severity of periodontitis in people with DM are significantly higher than in normoglycemic people. Although the correlation between DM and periodontitis is still not clear, it has been found that oxidative stress is a key pathogenic factor of diabetic periodontitis [131,132]. The role of DM in periodontitis has also been elucidated in diabetic rats. In fact, induction of periodontitis in diabetic rats led to a more severe alveolar bone loss and periodontal cell apoptosis than in normoglycemic rats. Moreover, in diabetic rats with induced periodontitis there was an increased local and systemic oxidative damage associated to a decreased NRF2 expression and increased levels of oxidative stress markers such as 3-NT-, 4-HNE-, MDA, and 8-OHdG in periodontal lesions. Thus, the enhanced local and systemic oxidative damage correlates to the downregulation of NRF2, favoring the development and progression of diabetic periodontitis [133].

Chronic periodontitis with diabetes mellitus (CPDM) has been correlated with the high glucose levels present in diabetic patients [134]. Moreover, CPDM onset is also favored by the increased ROS levels found in diabetic patients [135]. An interesting study found that human gingival epithelial cells (hGECs) treated with high glucose concentrations or *P. gingivalis* LPS have increased ROS levels while the mRNA levels of NRF2, catalase (CAT), glutamate-cysteine ligase catalytic subunit (GCLC), superoxide dismutase 1 (SOD1), and SOD2 were decreased [136]. However, ROS levels decreased after pretreatment with *baicalein* (BCI), a natural polyphenolic flavonoid that can be found in *Scutellaria baicalensis* Georgi (a flowering plant) [137]. In addition, BCI promoted the nucleus translocation of NRF2 inducing the expression of its target gene (CAT, GCLC, SOD1, and SOD2). Finally, a CPDM rat model treated with BCI showed an increased expression of NRF2 in periodontal tissue and mitigated the alveolar bone loss. Thus, BCI treatment may have beneficial effects in CPDM patients [136].

Diabetes plays also a key role in cell senescence, altering tissue repair. In fact, it has been reported that a high glucose microenvironment induces hPDLSC senescence, and senescent hPDLSCs show a diminished abilities to proliferate and differentiate impairing periodontal tissue repair and regeneration ability [138]. However, activation of NRF2/KEAP1 signaling can prevent cell senescence [139]. In a high glucose microenvironment, KEAP1 expression was increased while NRF2, HO-1, and NQO1 expression were significantly decreased, leading to increased oxidative stress [138]. Thus, therapies targeting NRF2 activation can reduce cell senescence, favoring periodontal tissue repair and regeneration in periodontitis by favoring in osteoblast or cementoblast hPDLSCs differentiation.

Uncontrolled DM leads to the accumulation of advanced glycation end-products (AGEs) through glycation reaction, causing several DM-associated complications such as diabetic retinopathy, peripheral neuropathy, and peripheral vascular diseases [140]. AGEs levels are significantly increased type-2 DM patients with chronic periodontitis compared to healthy individuals with or without periodontitis [141]. Moreover, AGEs trigger ROS accumulation, increasing oxidative stress and inflammation [142,143,144].

*Magnolol*, a natural compound found in *Magnolia officinalis*, showed protective effects in AGEs-exposed HGF, reducing ROS and increasing NRF2 and HO-1 expression. Moreover, magnolol significantly reduced AGEs-induced IL-6 and IL-8 production, demonstrating that magnolol has anti-inflammatory and antioxidant effects in AGEs-exposed HGF [145].

Thus, baicalein and magnolol may be used as a potential therapeutic approach for treatment of diabetes-associated periodontitis.

**Figure 4:**
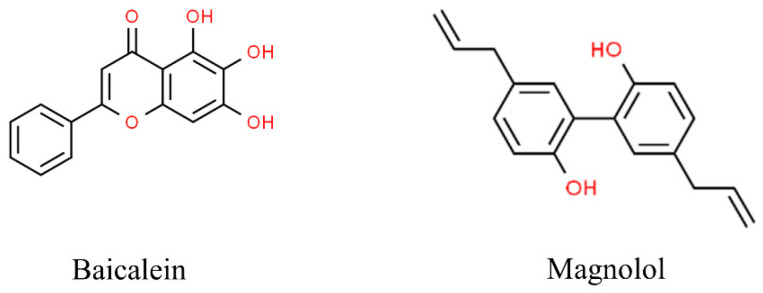
Molecular structures of baicalein and magnolol. The chemical structures of the compounds illustrated in this figure have been taken from ChemSpider free database (https://www.chemspider.com (accessed on 30 September 2024)).

The molecular structures of the natural compounds described above are shown in Figure 4.

## 5. Conclusions and Further Remarks

Periodontitis is a microbially-associated disease characterized by a host-mediated inflammation leading to the activation of host-derived proteinases, that result in loss of marginal periodontal ligament fibers, apical migration of the junctional epithelium, and apical spread of the bacterial biofilm along the root surface. Its progression depends on dysbiotic ecological modifications in response to nutrients from gingival inflammation and tissue damage.

The NRF2/KEAP1 signaling pathway is a promising target for future research since it can significantly improve several cellular processes. In this review, we discussed many studies highlighting the multifaceted role of this pathway in regulating many important processes, including inflammation and osteogenesis, and how this pathway can be modulated by a variety of natural compounds. In particular, it has been highlighted that certain compounds can reduce inflammation by inhibiting NF-κB signaling activation, a key pathway involved in cytokine production [93], thus reducing cytokine levels. In particular, the inhibition of NF-κB signaling activation was due to the increased HO-1 activity (a NRF2-dependent enzyme). In fact, it has been reported that this inhibitory effect of HO-1 on NF-κB signaling activation may be due to the products derived from HO-1 activity, such as CO [97].

The compounds discussed in this review activate NRF2/KEAP1 signaling, favoring the expression of several antioxidant enzymes that significantly reduce oxidative stress in the periodontium. The activation of this pathway also favors osteogenesis by inhibiting RANKL-induced osteoclast formation, thus inhibiting alveolar bone loss (as demonstrated in the in vivo model of periodontitis).

**Figure 5:**
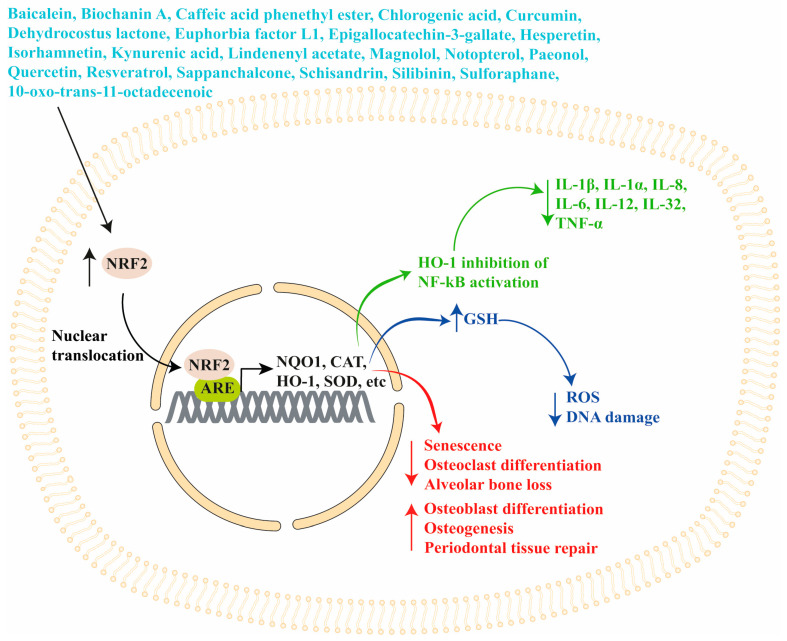
Modulation of NRF2/KEAP1 signaling by phytotherapeutics in periodontitis. Phytotherapeutics (in light blue) increase NRF2 expression, favoring its nuclear translocation and binding to the ARE regions present in the promoter of antioxidant genes (NQO1, CAT, HO-1, SOD, etc.), inducing their transcription. The increased expression of HO-1 inhibits NF-κB activation, thus reducing inflammatory cytokines production (in green). The activation of NRF2 also increases GSH levels, reducing ROS levels and DNA damage (in blue). Moreover, the activation of NRF2 reduces cell senescence, osteoclast differentiation, and alveolar bone loss while favoring osteoblast differentiation, osteogenesis, and periodontal tissue repair (in red). ARE, antioxidant response element; CAT, catalase; GSH, glutathione; HO-1, Heme-oxygenase 1; IL, interleukin; NQO1, NAD(P)H:quinone oxidoreductase; NRF2, Nuclear Factor Erythroid 2-Related Factor 2; SOD, superoxide dismutase; TNF-α, Tumor Necrosis Factor-α.

Overall, NRF2/KEAP1 signaling can be modulated by several natural compounds that could be used in combination with classical periodontitis treatments. In addition, since these compounds are safe for human consumption, they could be used as preventive treatment during gingivitis, since the worsening of this pathology leads to periodontitis (Figure 1). A schematic representation of NRF2/KEAP1 signaling modulation by phytotherapeutics in periodontitis is shown in Figure 5.

Since NRF2 activation plays a key function on protecting cells from oxidative stress and inflammation, as well as alveolar bone loss, the development of specific drugs or food supplements based on the natural compounds with this function may have a significant clinical impact not only in periodontitis treatment but also in the prevention of this disease.

It deserves to be pointed out that all the studies discussed in this review have been performed on cell lines or animal periodontitis models (mice and rats). Thus, specific clinical trials are necessary to evaluate the dose/effect relationship in humans in order to evaluate the beneficial effects of these phototherapeutics in patients with periodontitis.

## Data Availability

Data sharing is not applicable to this article as no datasets were generated or analyzed during the current study.
